# The Hypertriglyceridemic Waist Phenotype Is Associated with Several Cardiovascular Risk Factors in Women with Rheumatoid Arthritis

**DOI:** 10.3390/healthcare11030405

**Published:** 2023-01-31

**Authors:** Guadalupe Mendoza-Vázquez, Sandra Guzmán-Silahua, Jorge I. Gamez-Nava, Laura Gonzalez-Lopez, Mario Salazar-Paramo, Francisco Espinoza-Gómez, Carlos Riebeling-Navarro, María Claudia Espinel-Bermúdez, Arnulfo Hernán Nava-Zavala

**Affiliations:** 1 Unidad de Investigación Epidemiológica y en Servicios de Salud, Centro Médico Nacional de Occidente, Organo de Operacion Administrativa Desconcentrada, Instituto Mexicano del Seguro Social (IMSS), Guadalajara 44340, Jalisco, Mexico; 2 Programa de Doctorado en Ciencias Médicas, Facultad de Medicina, Universidad de Colima, Colima 28040, Colima, Mexico; 3 Programa de Doctorado en Farmacología, Centro Universitario de Ciencias de la Salud, Universidad de Guadalajara, Guadalajara 44340, Jalisco, Mexico; 4 Departamento de Fisiología, Instituto de Terapéutica Experimental y Clínica, Centro Universitario de Ciencias de la Salud, Universidad de Guadalajara, Guadalajara 44340, Jalisco, Mexico; 5 Departamento de Salud Pública, Centro Universitario de Ciencias de la Salud, Universidad de Guadalajara, Guadalajara, 44340, Jalisco, Mexico; 6 Academia de Inmunología, Departamento de Fisiología, Centro Universitario de Ciencias de la Salud (CUCS), Universidad de Guadalajara (UdG), Edificio O, Piso 1, Guadalajara 44340, Jalisco, Mexico; 7 Unidad de Investigación en Epidemiología Clínica, UMAE HP, Centro Médico Nacional SXXI, IMSS, Mexico City 06720, Mexico; 8 Unidad de Investigación Biomédica 02, UMAE, Hospital de Especialidades, Centro Médico Nacional de Occidente, Instituto Mexicano del Seguro Social (IMSS), Guadalajara 44340, Jalisco, Mexico; 9 Programa Internacional, Facultad de Medicina, Universidad Autónoma de Guadalajara, Zapopan 45129, Jalisco, Mexico; 10 Departamento de Inmunología y Reumatología, Hospital General de Occidente, Secretaría de Salud Jalisco, Zapopan 45170, Jalisco, Mexico

**Keywords:** hypertriglyceridemic waist, cardiovascular risk factors, rheumatoid arthritis, secondary prevention, tertiary prevention, tertiary healthcare

## Abstract

Rheumatoid arthritis (RA) associates with cardiovascular risk factors (CVRF) such as dyslipidemias and systemic inflammation. Cardiovascular Disease (CVD) is the leading cause of mortality. The hypertriglyceridemic waist phenotype (HTWP) identifies increased CVRF; however, information about HTWP on RA is scarce. Objective: To evaluate the association of HTWP with CVRF in RA. Material and methods: Cross-sectional study. Women (125) with RA were included (ACR, 1987). Anthropometry, bioimpedance, body mass index (BMI), disease activity score 28 (DAS28), and health assessment questionnaire disability index (HAQ-Di) were determined. The lipid profile determination includes the atherogenic index (AI) (TC/HDL) and Framingham Risk Score. HTWP is defined as a waist circumference ≥88 cm and triglycerides ≥ 150 mg/dL. Chi-squared and Student’s *t*-tests were applied for comparisons. Results: HTWP was found in 38 (30.4%) patients. The subgroup with HTWP had a greater frequency of arterial hypertension (AHT) (57.9 vs. 37.9, *p* = 0.04), Type 2 DM (23.7 vs. 8.0, *p*= 0.02), BMI (29.7 ± 3.2, vs. 26.8 ± 4.3, *p* < 0.001), fat mass (39.3 ± 4.8 vs. 34.7 ± 6.8, *p* < 0.001), and AI (4.7 ± 1.2 vs. 3.7 ± 1.0, *p* < 0.001). No differences between DAS28 and HAQ-Di were found. HTWP was associated with the presence of MetS and CVR (*p* < 0.001 and *p* = 0.012, respectively). Conclusion: The HTWP in RA is associated with CVRF, and its potential predictive role should be evaluated in longitudinal studies.

## 1. Introduction

Rheumatoid arthritis (RA) is an inflammatory, chronic, progressive disease of unknown etiology. It is characterized by the production of auto-antibodies, synovial inflammation, and hyperplasia, which can produce irreversible damage to the cartilage, bones, and articular and periarticular structures [1,2,3]. The prevalence of RA in adults ranges between 0.3% and 1% [4]. Extra-articular manifestations are associated with increased morbidity and mortality in these patients. Between 30 and 60% of cardiovascular disease (CVD) has been found in RA patients, explaining more than 50% of premature deaths in this population [1,2,3,4,5,6]. Classical cardiovascular risk factors (CVRF) such as obesity, dyslipidemia, type 2 diabetes mellitus (Type 2 DM), metabolic syndrome (MetS), arterial hypertension (AHT), familial antecedents of CVD, unhealthy lifestyle, and advanced age contribute to CVD in RA patients. Other factors could include premature atherosclerosis, the side effects of the treatment, alterations in body composition, and the chronic inflammatory stage [5,6,7]. Inflammatory biomarker levels frequently increase in patients with MetS and chronic diseases such as RA. Similarly, hypertriglyceridemia is a risk factor for developing cardiometabolic diseases, as dyslipidemia is among the main components of MetS [8]. The waist circumference (WC), although well correlated with the accumulation of abdominal visceral adipose tissues, does not suffice to identify abdominal obesity caused by excess visceral adipose tissue [9,10,11]. It should be mentioned that only a few CVRFs, such as the atherogenic index (AI), have been used as CVD prediction tools in RA patients [2,3]. The AI is the TC/HDL ratio and is categorized as low risk < 4.5, moderate risk 4.5–7, and high risk > 7 [2,3,12]. In the study of Castelli, the best performer as a cardiovascular risk factor estimator was the composite index, which includes the cardiovascular risk factors considered in the Framingham Risk Score. That seems logical, as coronary heart disease is a multifactorial disease; however, of all the factors, the lipids are the most fundamental, and mainly when they are expressed as AI [12].

Abdominal adiposity and dyslipidemia are associated with developing MetS, higher atherogenic tendency, and insulin resistance (IR). The combination of hypertriglyceridemia and large WC is called the hypertriglyceridemic waist phenotype (HTWP). The HTWP performs as a simple and economical clinical tool to identify individuals with excess adipose tissue, visceral obesity, increased levels of insulin, apolipoprotein B, small and dense LDL particles, and chronic inflammation [8,13,14]. In turn, the HTWP allows for the follow-up of asymptomatic individuals with cardio-metabolic risk factors. Hence, it is beneficial in clinical practice and prevention and health promotion strategies [9,10,11] because HTWP is a tool associated with CVRF, as those present in RA. This study aimed to describe the frequency of HTWP and its association with other CVRFs.

## 2. Materials and Methods

### 2.1. Study Design

This is a cross-sectional study.

### 2.2. Study Population

The study included 125 female patients (≥18 years of age) that attended the Unidad de Investigación Biomédica 02, UMAE, Hospital de Especialidades Centro Médico Nacional de Occidente, Instituto Mexicano del Seguro Social (IMSS). Guadalajara, Jalisco, México fulfilled the 1987 classification criteria of the American College of Rheumatology for RA [1]. The inclusion period was 26 months, from March 2014 to May 2016. The value of the disease activity index was determined in 28 joints (DAS28, disease activity score 28) [15], with a values score of 0 to 10, where: <2.6 remissions; 2.6–3.19 low activity; 3.2–5.09 moderate activity; and >5.1 high activity [15]. The erythrosedimentation rate (ESR) was determined with the Wintrobe method, and the C-reactive protein (CRP) was determined through nephelometry (Behring Nephelometer™ II Analyzer, BNII, Siemens, Marburg, Germany) and considered as a component of DAS28. Disability was measured using the HAQ-Di (health assessment questionnaire disability index); the questionnaire consisted of eight functionality domains: getting dressed, getting up, eating, walking, hygiene, outreach, grip, and normal activities; each question was scored from 0 to 3, representing 0 (without difficulty), 1 (some difficulty), 2 (much difficulty), and 3 (unable to perform it). The highest scores of each domain were averaged in a global HAQ Di score on a scale of 0.0 to 0.3 [16]. The clinical charts were reviewed for disease-modifying drugs (DMDs). The DMDs registered were chloroquine, leflunomide, methotrexate (MTX), biological agents, glucocorticoids, and non-steroidal anti-inflammatory drugs (NSAIDs). Those patients with other rheumatic diseases, chronic infections, renal failure, psychiatric disease, cancer, thyroid disease, and pregnancy were excluded. The evaluation of exclusion criteria relied on the information obtained when reviewing the clinical record of each patient.

### 2.3. Determination of Serological Parameters

A sterile antecubital vein puncture was conducted using Vacutainer^®^ tubes without anticoagulants. The sample was then centrifuged at 3500 rpm for 15 min at room temperature and aliquoted in micro vials. Blood samples came from 12 h fasted patients in the morning. All serum samples were stored at −20 °C for ulterior determination in the reference laboratory. 

The rheumatoid factor (RF) was determined through nephelometry (Behring Nephelometer™ II Analyzer, BNII, Siemens, Marburg, Germany). Analyses included Total cholesterol (TC), high-density lipoprotein (HDL), and triglycerides (TG). The Friedwald formula [LDL = TC – HDL − TG/5] was used to determine low-density lipoprotein (LDL) [17]. 

Glucose was determined with the glucose oxidase technique (VITROS^®^ 4600 System, Ortho-Clinical Diagnostics, Inc, Rochester, NY, USA) [18]. Type 2 DM diagnosis was considered if patients had a previous diagnosis, were under antidiabetic treatment, or had a fasting glucose value of ≥126 mg/dL [19].

### 2.4. Arterial Pressure

Arterial pressure was measured with a mercury sphygmomanometer, using an inflatable cuff adequate for the thickness of the left arm of each patient, after a resting time of 20 min, considering as abnormal values a systolic pressure (SP) ≥ 140 mmHg and a diastolic pressure (DP) ≥ 90 mmHg [20].

### 2.5. Anthropometric Evaluation

Anthropometric determinations were performed according to the criterion of the international society for the Advancement of Kinanthropometry (ISAK) [21].

Body weight was determined in kilograms (kg) in barefoot patients with light clothes using a bioimpedance scale (TANITA- BC-533) with a 0 to 150 kg scale, with an induction frequency of 50 kHz [22]. It was determined with a 1-mm accuracy using a Seca^®^ model 206 stadiometer with the head of the patient in the Frankfurt plane [21]. After calibrating the instrument, patients stand on the scale with their arms relaxed beside their trunk for foot-foot (metatarsus-calcareous) measurement [21,22,23]. The body mass index (BMI) was calculated according to the Quetelet formula [BMI = weight (kg)/height (m)^2^] [23]. The classification proposed by the World Health Organization (WHO) was used (normal weight BMI > 18.5 kg/m^2^, <24.9 kg/m^2^; overweight BMI ≥ 25 kg/m^2^, <25.9; and obesity BMI ≥ 30 kg/m^2^) [23]. The WC was measured at the midpoint between the last rib and the iliac rest at the end of an expiration with a flexible metric Lufkin^®^ tape, with patients standing with crossed arms followed by a relaxed position [21]. 

### 2.6. Metabolic Syndrome

The MetS was determined when the patient fulfilled three or more of the five criteria of the National Cholesterol Education Program Adult Treatment Panel III: WC ≥ 88 cm; TG ≥ 150 mg/dL; HDL < 50 mg/dL; blood pressure ≥ 130/≥85 mmHg; fasting glucose ≥ 110 mg/dL [24]. 

### 2.7. HTWP

The HTWP construct was defined according to the cut-off points proposed by the NCEP-ATP III for women, which refers to a WC of 88 cm or more, as well as TG levels of 150 mg/dL or more. Patients were stratified into two groups: (a) without HTWP; or (b) with HTWP (WC ≥ 88 cm + TG ≥ 150 mg/dL) [9].

### 2.8. Cardiovascular Risk by Atherogenic Index

For this work, the TC/HDL ratio was used as a dichotomic estimation of the CVR, considering a low CVR of <4.5 and a high CVR of ≥4.5. It was calculated by the Framingham Risk-Score considering a low risk < 10 and intermediate or high risk >10 [12,25].

### 2.9. Statistical Analysis

Qualitative variables were expressed in proportions and were compared with the Chi-square test or Fisher’s exact test. Quantitative variables were expressed with a mean ± standard deviation (SD) and were compared with a Student’s *t*-test for independent samples. A value was considered significant at *p* ≤ 0.05. 

Bivariate and multiple logistic regression models explored the relationships between HTWP and CVRF. The odds ratio was expressed with an upper and lower confidence limit of 95% and significance values for Mantel-Haenszel and Cox-Snell for Pseudo-R-Square. The software SPSS^®^ version18.0 for Windows (SPSS Inc. Chicago, IL, USA) was used.

### 2.10. Ethical Considerations

This project was approved by the Ethics and Research Committee of the Mexican Social Security Institute (IMSS, for its initials in Spanish). This study followed the ethical principles of the Declaration of Helsinki. All patients signed a written consent once informed verbally and in writing of the characteristics and scope of the study. 

## 3. Results

The study included 125 female patients with the following comorbidities: Type 2 DM in 16 (12.8%), AHT in 55 (44%), and MetS in 61 (48.8%). Table 1 describes other general characteristics. By stratifying the groups according to HTWP, 38 (30.4%) were positive, and 87 (69.6%) were negative. 

Comparing these two groups (Table 1) revealed significantly elevated values in the HTWP group for body weight, BMI, fat mass, WC, AHT, Type 2 DM, MetS, TC, TG, and CVR. The frequency of high CVR was higher in the group with HTWP (44.7% vs. 24.1%, *p* < 0.001) and, as expected, the frequency of FRS ≥ 10 was also higher in the HTWP group (71.1% vs. 40.2%, *p* = 0.02). Remarkably, smoking did not reach a significant difference between groups with and without HTWP.

Among the clinical characteristics of the 125 RA patients (Table 2), the duration of the disease in years was 13.2 ± 9.4; the DAS28 was 4.0 ± 1.2. Regarding pharmacological therapy, 119 (95.2%) received DMDs, and 94 (75.2%) received glucocorticoids. When comparing the groups with and without HTWP, no significant differences were found for disease duration, HAQ-Di and DAS28 (Table 2). Regarding pharmacological treatment, a higher frequency of MTX use (*p* = 0.02) was found in patients without HTWP.

The bivariate model showed relationships between HTWP and T2 DM, metabolic syndrome, overweight or obesity, fat mass, TC, FRS, CVRF, FRS, and methotrexate (Table 3), and the final multiple logistic regression showed that HTWP was associated with the presence of MetS and CVR.

## 4. Discussion

In our RA patients, the body weight was similar to that reported in other studies [26,27] in Mexican (63.3 ± 11.8 kg) and Iranian patients (66.9 ± 12 kg). BMI values (27.7 ± 4.2 kg/m^2^) indicate overweight in Mexican patients according to other studies [26,28], and they were found to range from 26.9 ± 4.5 kg/m^2^ to 28.6 ± 5.6 kg/m^2^. 

The accumulated 76% frequency of overweight and obesity obtained in our study is similar to the 73% obtained in adult women in the National Survey of Health and Nutrition 2012 (ENSANUT 2012, for its initials in Spanish), and that of 75.6% in adult women identified in the National Survey on Health and Nutrition Half Way 2026 (ENSANUT MC 2016) [29,30]. In the international literature, the frequency of the overweight and obesity combination ranges from 29.2% to 80.3% [26,31,32,33].

Regarding the MetS and AHT components, these were found in 44% of our patients, finding reports from 8.1% to 60.7% [27,28,31,34,35,36,37]. The results of AHT observed in our patients are relevant because, according to ENSANUT 2012 data, women present this entity in 30.8% of cases [29], whereas, in the ENSANUT MC 2016, the prevalence of AHT is 26.1%, of which only 70.5% had a previous medical diagnosis [30]. In addition, the total proportion of adults with a previous AHT diagnosis with controlled blood pressure values (<140/90 mmHg) is 58.7% according to the criteria of the American Heart Association [30]. We observed a relative frequency between HTWP and AHT (*p* = 0.02), which could be due to a set of factors that leads to the development of this ailment, such as age, high BMI, DM, dyslipidemia, and high TG [20].

In our study, Type 2 DM was found in 12.8% of participants, similar to previous studies performed in Mexico (12.1%) [35] and Croatia (10.3%) [37]. Notwithstanding this, other authors have reported frequencies from 3.8% to 9.7% [27,28,31,34]. The Type 2 DM frequency reported in our patients is similar to that found in Mexican women (14.4%) according to data from ENSANUT 2012 [29]. It should be noted that the highest increase in Type 2 DM prevalence when comparing ENSANUT 2012 with ENSANUT 2016 was observed among women aged 60 years and over (a range considered in the SD of our study) [30]. 

Regarding the WC values of our patients (92.1 ± 10.4 cm), these are equivalent to those described in previous reports (92.3 ± 14 cm; 93 cm (53 to 124), 92 cm (84 to 100), and 90 cm (80 to 99) [27,37,38,39]. When comparing patients with or without HTWP, the WC showed significant differences (*p* < 0.001). 

We found an increased frequency in MetS of 84.8%. In contrast, another study in a population similar to ours reported an increased frequency in MetS of 94% [37], despite our total population only being female patients (who tend to accumulate a higher percentage of MetS).

Our study shows a significant bivariate association of HTWP with Type 2 DM, fat mass ≥ 30%, and MetS (*p* = 0.03, *p* = 0.02 and *p ≤* 0.001, respectively). These associations may result from a central pattern with body fat distribution combined with higher visceral fat, leading to an independent risk factor for developing Type 2 DM. The latter means that those subjects with an excess of intra-abdominal or visceral adipose tissue will be at greater risk of developing IR and other characteristics of MetS [9,10]. 

Due to the increase in MetS, it is now the most frequent nutritional alteration in the adult population according to data from ENSANUT MC 2016 (defined by the survey as excess weight resulting from overweight and obesity) [30]. Our literature review found that the MetS frequency in RA reaches 61% [27,31,33,34,36,37,38,39,40,41]. In our study, a prevalence for MetS of 58.8% was observed, which lies within the mentioned range, and is more similar to that reported by Sahebari et al. and Zaragoza-García et al. [27,41]. The reviewed studies and the mentioned prevalences were considered based on the criteria of the NCEP ATP III [27,33,34,36,37,38,39,40]. The multiple regression analysis identified the association of HTWP with the presence of MetS and TC (*p ≤* 0.001 and *p =* 0.01, respectively). These results suggest the latter’s relevance in RA, as it strengthens its relationship with MetS and lipid abnormalities. 

Regarding our findings on the lipids profile, the mean values and the SD of TG in RA (147.1 ± 70.0 mg/dL) differ from those reported by other authors (105 ± 36 mg/dL) [27] and (171.9 ± 57.2 mg/dL) [33]. On the other hand, other authors have reported different TG values (145 mg/dL (30 to 186)) [39], (121 mg/dL (45 a 270)) [40], and (102 mg/dL (53 to 437)) [39] which do not agree with our results.

We observed a mean and SD for TG of 196.4 ± 37.8 mg/dL, a level that is not far from that reported by other authors (202 ± 180 mg/dL) [27], (188.9 ±33.2 mg/dL) [28], (173 mg/dL [83 a 290 mg/dL]) [38].

The TC/HDL ratio reported in this study (4 ± 1.2 mg/dL) was similar to another report (4.4 ± 1.1 mg/dL) [33] but was different from another previous study (3.6 ± 0.9 mg/dL) [28]; remarkably, this information came from Mexican patients with RA. 

The lipids profile of our results differs from the previously reported lipids profile. This difference could be attributed to pharmacological issues, such as hypoglycemic drugs or DMDs. 

Abdominal adiposity and dyslipidemia are the pathogenic core of MetS. The related measurement, the HTWP, could better identify those subjects with a greater atherogenic tendency [11]. Women with HTWP tend to have higher variation in TC levels, and a TC/HDL ratio compared to women without HTWP (*p* < 0.001, *p* < 0.001, and *p* < 0.001, respectively).

Previous studies reported an association between the improvement in TG levels and the use of MTX [26,32,34,35,37,39]. On the other hand, we found a relative frequency (*p* = 0.02) and a bivariate association between HTWP and the use of MTX (*p* = 0.035). A total of 57.6% of all our patients underwent MTX treatment, and 42.1% with HTWP were MTX-treated. In the reviewed literature, MTX treatment was as high as 75% [27,33,35,36,38,40]. 

The HTWP significantly bivariate associated with variables such as Type 2 DM, percentage of fat mass, MetS, TC, CVR, FRS, and MTX. 

In contrast, the isolated finding of large WC does not predict increased visceral adipose tissue or a higher risk of developing cardio-metabolic diseases. [8,10].

Similarly to another study [36], in this study we suggest a protective effect of MTX, which could be specific to the drug and may not result from an anti-inflammatory effect because it was not observed with any other DMD. Another similar characteristic of our study [36] concerning the protective effect of MTX in patients 60 years and older [35] is that our population’s mean age was 59 ± 10 years (which includes patients older than 60 years).

Regarding the disease activity evaluated by DAS28, no significant difference was found between the groups with and without HTWP. However, in a recent paper [42], the authors examined the time-varying association of the rheumatoid arthritis disease activity measured using the CDAI score (Clinical Disease Activity Index) to subsequent CVD. They found that having a higher CDAI level earlier during the follow-up may be more strongly associated with CVD than having the same CDAI level later during the follow-up among patients within five years of onset at the time of registry enrollment. Our work is unable to analyze this relationship class because of its cross-sectional design; however, exploring whether the HTWP could associate with this time-varying CDAI score and the cardiovascular outcome will be interesting. 

Our study has several limitations: (a) a cross-sectional design, which allows for establishing an association but not causality; the latter should be evaluated in longitudinal studies; (b) the lack of a control group of healthy individuals; (c) stratified analyses for therapeutic drugs; (d) the lack of inclusion of both genders; and (e) the lack of analysis for the erythrosedimentation rate (ESR) and C-reactive protein (CRP) values independent of the DAS28 construct and included correlation analysis. In addition, in clinical practice, the measurement of the metabolic triad and the accurate quantification of adipose tissue through imaging methods are of limited use because of their high cost and time investment; in contrast, the HTWP is easily obtained [8].

## 5. Conclusions

Our results indicate the association of HTWP with CVD predicting variables, supporting the clinical application of HTWP as an auxiliary tool for the detection and risk evaluation for CVD in RA, particularly in those patients that might partially fulfill the MetS criteria. HTWP can provide information on the presence of risk that the clinician could consider in establishing a preventive care/treatment plan for patients prone to cardiovascular-type diseases. Prospective studies are needed to strengthen the use of HTWP as a clinical and predictive tool and to explore the association of HTWP with both AI and FRS to evaluate if they share predictive properties for cardiovascular outcomes. The presented results should be extrapolated carefully to other populations because this study was performed exclusively on Mexican women with RA. 

## Figures and Tables

**Table 1:** Comparison of general characteristics according to the hypertriglyceridemic waist phenotype in 125 women with rheumatoid arthritis.

HTWP				
Variable	Present (*n* = 38)	Absent (*n* = 87)	Total (*n* = 125)	*p*
Clinical-demographic				
Age, years	59.2 ± 9.1	59.1 ± 10.8	59.1 ± 10.3	0.94
Weight, kg	70.5 ± 8.9	64.5 ± 11.2	66.3 ± 10.9	0.004
Height, m	1.5 ± 0.1	1.5 ± 0.1	1.5 ± 0.1	0.49
Physical activity, *n* (%)	8 (21.1)	31 (35.6)	39 (31.2)	0.11
Smoking, *n* (%)	9 (23.7)	12 (13.8)	21 (16.8)	0.17
Arterial hypertension, *n* (%)	22 (57.9)	33 (37.9)	55 (44.0)	0.04
Type 2 diabetes mellitus, *n* (%)	9 (23.7)	7 (8.0)	16 (12.8)	0.02
Metabolic syndrome, *n* (%)	36 (94.7)	25 (28.7)	61 (48.8)	<0.001
Body composition				
BMI, kg/m^2^	29.7 ± 3.2	26.8 ± 4.3	27.7 ± 4.2	<0.001
-Low weight, *n* (%)	0	2 (2.3)	2 (1.6)	0.49
-Normal, *n* (%)	1 (2.6)	27 (31.0)	28 (22.4)	<0.001
-Overweight, *n* (%)	25 (65.8)	39 (44.8)	64 (51.2)	0.03
-Obesity, *n* (%)	12 (31.6)	19 (21.8)	31 (24.8)	0.25
WC, cm	97.7 ± 7.0	89.7 ± 10.7	92.1 ± 10.4	<0.001
Fat mass, (%)	39.3 ± 4.8	34.7 ± 6.8	36.1 ± 6.6	<0.001
-Fat mass ≥ 30%, *n* (%)	37 (97.4)	69 (79.3)	106 (84.8)	0.01
Serological data				
Glucose, mg/dL	111.9 ± 55.2	96.8 ± 33.7	101.4 ± 41.8	0.13
TC, mg/dL	217.1 ± 38.6	187.4 ± 33.9	196.4 ± 37.8	<0.001
HDL, mg/dL	49.1 ± 14.9	53.3 ± 14.4	52.0 ± 14.6	0.14
LDL, mg/dL	113.3 ± 36.0	109.1 ± 30.2	110.4 ± 32.0	0.51
TG, mg/dL	218.9 ± 53.6	11 5.7 ± 50.6	147.1 ± 70.0	<0.001
AI, TC/HDL ratio	4.7 ± 1.2	3.7 ± 1.0	4.0 ± 1.2	<0.001
-CVR high ≥ 4.5, *n* (%)	19 (50.0)	21 (24.1)	40 (32.0)	<0.001
FRS ≥ 10, *n* (%)	27 (71.1)	35 (40.2)	62 (49.6)	0.02

Quantitative variables are expressed in means and standard deviation; quantitative variables are expressed in numbers and percentages. A Student’s *t*-test was used to compare means and the Chi-squared and Fisher’s exact test were also used. HTWP, hypertriglyceridemic waist phenotype; BMI, body mass index; WC, waist circumference; TC, total cholesterol, HDL, high-density lipoproteins; LDL, low-density lipoproteins; TG, triglycerides; AI, atherogenic index; FRS, Framingham Risk Score.

**Table 2:** Comparison of clinical characteristics according to the hypertriglyceridemic waist phenotype group.

Hypertriglyceridemic Waist				
Variable	Present (*n* = 38)	Absent (*n* = 87)	Total (*n* = 125)	*p*
Disease characteristics				
Evolution of the disease, years	12.2 ± 8.6	13.6 ± 9.7	13.2 ± 9.4	0.45
-Evolution > 10 years, *n* (%)	21 (55.3)	49 (56.3)	70 (56.0)	0.91
HAQ-Di	0.54 ± 0.1	0.56 ± 0.0	0.51 ± 0.55	0.95
DAS28, score	3.11 ± 0.9	3.2 ± 1.2	4.0 ± 1.2	0.79
-Remission, *n* (%)	14 (36.8)	33 (37.9)	47 (37.6)	
-Low activity, *n* (%)	11 (28.9)	24 (27.6)	35 (28.0)	
-Moderate activity, *n* (%)	11 (28.9)	24 (27.6)	35 (28.8)	0.99
-High activity, *n* (%)	2 (5.3)	6 (6.9)	8 (6.4)	0.95
Pharmacological therapy				
Glucocorticoids, *n* (%)	32 (84.2)	62 (71.3)	94 (75.2)	0.12
NSAIDs, *n* (%)	30 (78.9)	74 (85.1)	104 (83.2)	0.40
DmdS, *N* (%)	37 (97.4)	82 (94.3)	119 (95.2)	0.45
-Cloroquine, *n* (%)	3 (7.9)	11 (12.6)	14 (11.2)	0.44
-Leflunomide, *n* (%)	16 (42.1)	25 (28.7)	41 (32.8)	0.14
-Methotrexate, *n* (%)	16 (42.1)	56 (64.4)	72 (57.6)	0.02
Biological agents, *n* (%)	2 (5.3)	12 (13.8)	14 (11.2)	0.16

Quantitative variables are expressed in means and standard deviation; qualitative variables are expressed in numbers and percentages. A Student’s *t*-test was used to compare means, and Chi-squared and Fisher’s exact tests were also used. DAS28, disease activity score; HAQ-Di, health assessment questionnaire disability index; NSAIDs, non-steroidal anti-inflammatory drugs; DMDs, disease-modifying drugs.

**Table 3:** Logistic regression showing the odds ratio of the hypertriglyceridemic waist with cardiovascular risk variables.

Variables	Bivariate Regression Analysis				Multiple Logistic Regression			
	OR	95% CI.		*p*	OR	95% CI.		*p*
		Lower	Upper			Lower	Upper	
Arterial hypertension	2.25	1.03	4.88	0.06	-	-	-	-
Type 2 diabetes mellitus	3.54	1.21	10.39	0.03	-	-	-	-
Metabolic syndrome	44.64	9.98	199.6	<0.001	62.71	9.24	425.60	<0.001
Overweight or obesity	18.50	2.41	141.6	0.001	4.49	0.48	41.98	0.188
Fat mass ≥ 30%	9.65	1.23	75.19	0.02	-	-	-	-
Total cholesterol ≥ 200	4.66	2.03	10.68	<0.001	4.70	1.36	16.20	0.01
CVR high ≥ 4.5	3.14	1.40	7.01	<0.001	-	-	-	-
FRS ≥ 10	3.67	1.60	8.29	0.03	-	-	-	-
Methotrexate	2.48	1.13	5.41	0.03	-	-	-	-

OR as an Odds Ratio. Bivariate significance statistical values for Mantel-Haenszel. Pseudo-R-Square final model 0.40 for Cox-Snell.

## Data Availability

Non-applicable.
